# Case Report: A pediatric case of multifocal *Streptococcus intermedius* brain abscesses as the initial manifestation of SMAD4-associated juvenile polyposis–hereditary hemorrhagic telangiectasia overlap syndrome

**DOI:** 10.3389/fped.2026.1866003

**Published:** 2026-09-24

**Authors:** Nala Al-Khatib, Patricia Martin, Katherine Sinchek, Colleen Buhrfiend, Lubov Romantseva, Edith Chernoff, David C. Nguyen

**Affiliations:** 1 Department of Pediatrics, Rush University Medical Center, Chicago, IL, United States; 2 Department of Pediatric Pulmonology, Ann & Robert H. Lurie Children’s Hospital of Chicago, Chicago, IL, United States; 3 Department of Neurology, Rush University Medical Center, Chicago, IL, United States; 4 Department of Pediatric Neurology, Rush University Medical Center, Chicago, IL, United States; 5 Department of Pediatrics, Section of Genetics and Genomics, University of Chicago, Chicago, IL, United States; 6 Department of Pediatrics, Division of Pediatric Infectious Diseases, Rush University Medical Center, Chicago, IL, United States

**Keywords:** brain abscesses, hemorrhagic hereditary telangiectasia, juvenile polyposis hereditary hemorrhagic telangiectasia (JPHT), pediatrics, *Streptococcus intermedius*

## Abstract

Hereditary hemorrhagic telangiectasia (HHT) is a rare genetic disease characterized by epistaxis, telangiectasias, and arteriovenous malformations (AVMs). Clinical diagnosis of HHT is typically guided by fulfillment of the Curaçao criteria. HHT is a known risk factor for brain abscesses in the pediatric population, particularly in those with pulmonary AVMs, where intrapulmonary right-to-left shunting permits septic emboli to seed the brain. We report the case of a 9-year-old girl with asthma and chronic sinusitis who presented with 5 days of congestion, cough, and worsening headache in the setting of Coronavirus Disease 2019 infection. Due to persistent hypoxia and transient altered mental status, further evaluation was pursued, with brain magnetic resonance imaging revealing multiple ring-enhancing lesions and chest computed tomography (CT) demonstrating multiple pulmonary nodules. CT angiography confirmed diffuse pulmonary AVMs. Culture from a brain biopsy grew *Streptococcus intermedius*. Whole-genome sequencing identified a *de novo* heterozygous *SMAD4* variant, supporting a molecular diagnosis of juvenile polyposis/HHT syndrome. This case illustrates an atypical presentation and early diagnosis of this syndrome, prompted by the incidental finding of multiple subclinical brain abscesses. It underscores the value of maintaining a broad differential and performing targeted diagnostic studies when encountering brain abscesses of unclear origin. Pulmonary AVMs should be considered as a potential etiology of brain abscess, particularly in patients with concomitant hypoxemia and when endocarditis has been excluded. Furthermore, genetic evaluation for HHT should be considered in pediatric patients with unexplained or multifocal brain abscesses, even when the Curaçao criteria are not met, as early recognition may help prevent neurologic complications.

## Introduction

Hereditary hemorrhagic telangiectasia (HHT), also known as Rendu–Osler–Weber disease, is a rare autosomal dominant genetic disorder characterized by recurrent epistaxis, mucocutaneous telangiectasias, and visceral arteriovenous malformations (AVMs). Pulmonary AVMs (PAVMs) are a known risk factor for brain abscesses, in which intrapulmonary right-to-left shunting allows septic emboli to bypass pulmonary filtration and seed the brain (1–5). Because clinical manifestations often develop gradually over decades, many individuals with HHT remain undiagnosed until later in life. Importantly, a subset of patients with HHT may also present with juvenile polyposis (JP), an autosomal dominant disorder characterized by hamartomatous gastrointestinal (GI) polyps that typically present during childhood. This combined condition, described as JP/HHT overlap syndrome, is a rare condition associated with the *SMAD4* gene, encompassing varying clinical manifestations of both diseases (6).

*Streptococcus intermedius*, a member of the *Streptococcus anginosus* group (SAG), is a common pathogen in pediatric brain abscesses, most often arising from contiguous spread associated with sinusitis or other infections of the nasal or oral cavities (7, 8). Less commonly, it has been seen in cases involving hematogenous dissemination from distant sites such as pulmonary infections, intracardiac or intrapulmonary shunting, and endocarditis (9, 10). Multifocal or hemorrhagic presentations are distinctly uncommon (7–10).

We present the case of a 9-year-old with chronic sinusitis who was found to have multifocal intracranial abscesses secondary to *S. intermedius*, ultimately leading to an underlying diagnosis of JP/HHT overlap syndrome, identified not by clinical criteria but through genetic testing.

## Case description

A 9-year-old girl with mild intermittent asthma, chronic rhinosinusitis, and recurrent otitis media requiring bilateral tympanostomy tubes presented with 5 days of nasal congestion, cough, and headache. On examination, she was tachycardic (124 beats per minute), tachypneic (32 breaths/min), low-grade febrile (100.1 °F), and hypoxemic (SpO_2_ 89% on room air). Due to persistent respiratory distress and hypoxemia despite bronchodilators, she was transferred to the emergency department for escalation of care.

Initial evaluation revealed mild leukocytosis (10.40 × 10^3^ cells/µL) and elevated C-reactive protein (67 mg/L) with an unremarkable chest X-ray. Computed tomography (CT) of the brain, obtained for persistent headache, demonstrated multifocal hypodense lesions in the right frontal and occipital lobes, along with opacification of the right paranasal sinuses and bilateral mastoid effusions. A SARS-CoV-2 PCR test returned positive. She was admitted to the Pediatric Intensive Care Unit (PICU), where physical exam additionally revealed digital clubbing.

Overnight, she experienced a brief transient episode of decreased responsiveness, prompting a repeat CT head with contrast, which showed multiple ring-enhancing lesions with surrounding edema, with magnetic resonance imaging (MRI) demonstrating restricted diffusion, suggesting possible abscesses (Figure 1). Magnetic resonance angiography (MRA) and venography (MRV) demonstrated patent cerebral blood vessels. CT showed no bony erosion, and MRI of the temporal bones revealed no fistulous intracranial communication. Otolaryngology was consulted, and nasal endoscopy found normal nasal mucosa.

**Figure 1:**
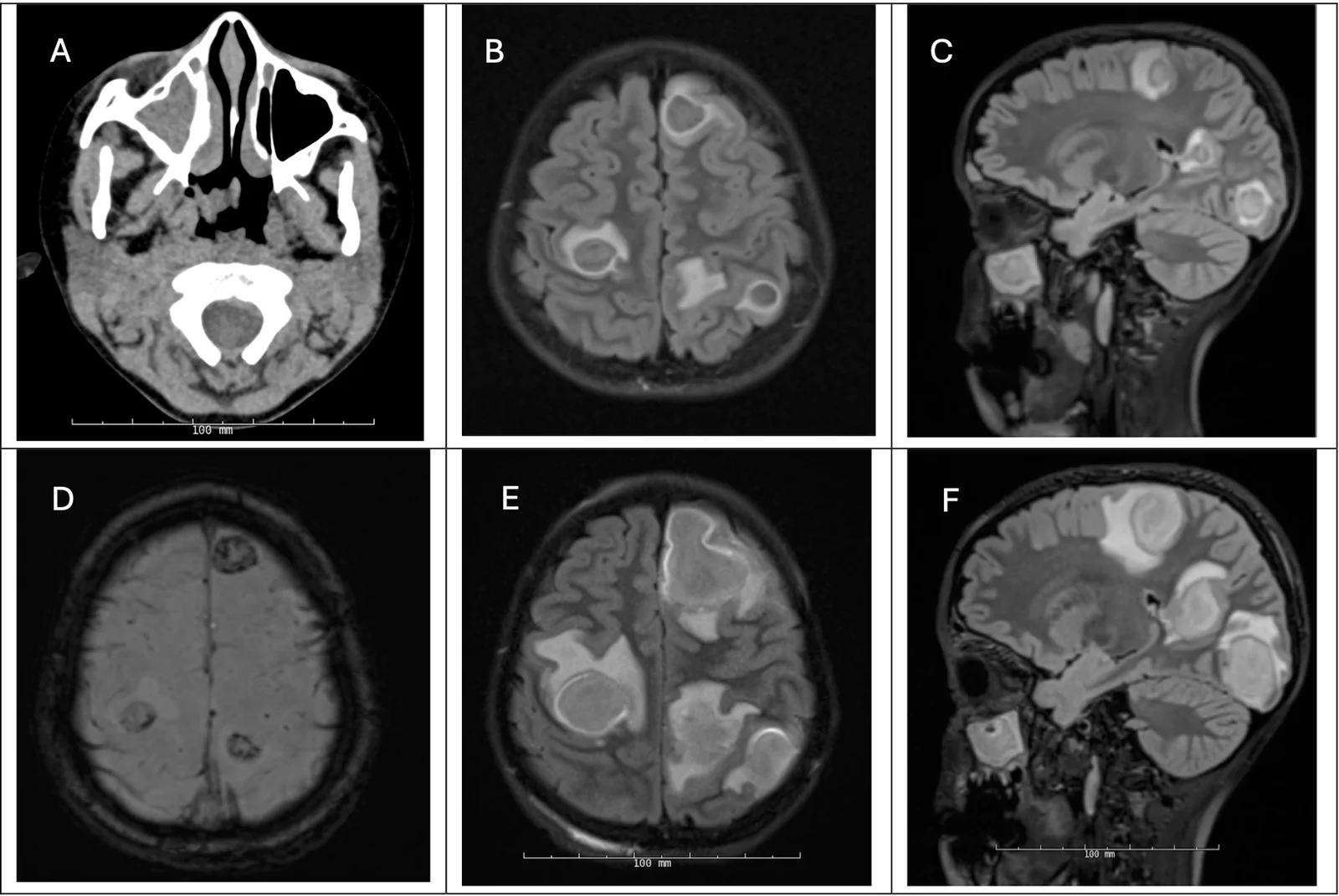
Head imaging. **(A)** Initial head CT (axial view) on hospital day 1 highlighting bilateral mastoid effusions and near-complete opacification of the right paranasal sinuses. **(B)** Initial MRI T2 FLAIR (axial view) highlighting multiple small ring-enhancing lesions in both cerebral hemispheres with vasogenic edema. **(C)** Initial MRI T2 FLAIR (sagittal view) highlighting multiple lesions. **(D)** MRI SWI (axial view) highlighting hemorrhagic component of lesions. **(E,F)** Repeat MRI T2 FLAIR (axial and sagittal, respectively) on hospital day 10 following transient discontinuation of steroids, highlighting increased size of multiple lesions and surrounding edema.

A lumbar puncture was performed. The opening pressure was elevated (29 cm H_2_O), and the cerebrospinal fluid did not demonstrate pleocytosis. A broad infectious and non-infectious workup was done, ultimately returning negative (Table 1). Transthoracic echocardiography found no evidence of valvular vegetations or intracardiac shunting. Chest CT revealed innumerable bilateral ground-glass and nodular opacities (Figure 2). Abdominal and pelvic CT scans were unremarkable. The patient underwent burr-hole biopsy of a peripheral brain lesion, yielding frank pus. Cultures from the brain biopsy grew *S. intermedius,* which was also isolated from her nasal endoscopy specimen.

**Table 1:** Diagnostic laboratory and microbiologic evaluation.

Specimen/source	Key findings
Blood/serum	Negative bacterial, mycobacterial, and fungal cultures
	Negative serology for *Blastomyces*, *Histoplasma*
	Negative HIV antibody/antigen
	Negative cryptococcal antigen
	Negative *Toxoplasma gondii* IgG and IgM
	Negative *Bartonella* serology
	Negative Q fever serology
Bronchoalveolar fluid	Negative multiplex respiratory pathogen panel
	Negative bacterial, mycobacterial, and fungal cultures
	Negative *Pneumocystis* smear negative
	Negative cytology for malignancy
Nasopharyngeal swab	SARS-CoV-2 PCR positive
Nasal tissue	Culture positive for *S. intermedius*
Urine	Blastomyces antigen negative
Brain abscess (right parietal lesion)	Aerobic culture positive for *S. intermedius*
	Pathology: necroinflammatory debris with acute inflammation, consistent with abscess
Cerebrospinal fluid	Negative bacterial, mycobacterial, and fungal cultures
	Negative cryptococcal antigen
	Negative HSV-1/2 and VZV DNA PCR
	Negative cytology for malignant cells
Non-infectious workup	Immunologic: Quantitative immunoglobulins and TBNK (T, B, and Natural Killer cells) panel unremarkable
	Autoimmune: CSF: negative myelin oligodendrocyte glycoprotein (MPO), oligoclonal bands, and aquaporin-4 antibodies
	Serum: negative antinuclear antibody (ANA), anti-neutrophil cytoplasmic antibodies (ANCA), and serum protein electrophoresis (SPEP)
	Urine: negative urine protein electrophoresis (UPEP)

**Figure 2:**
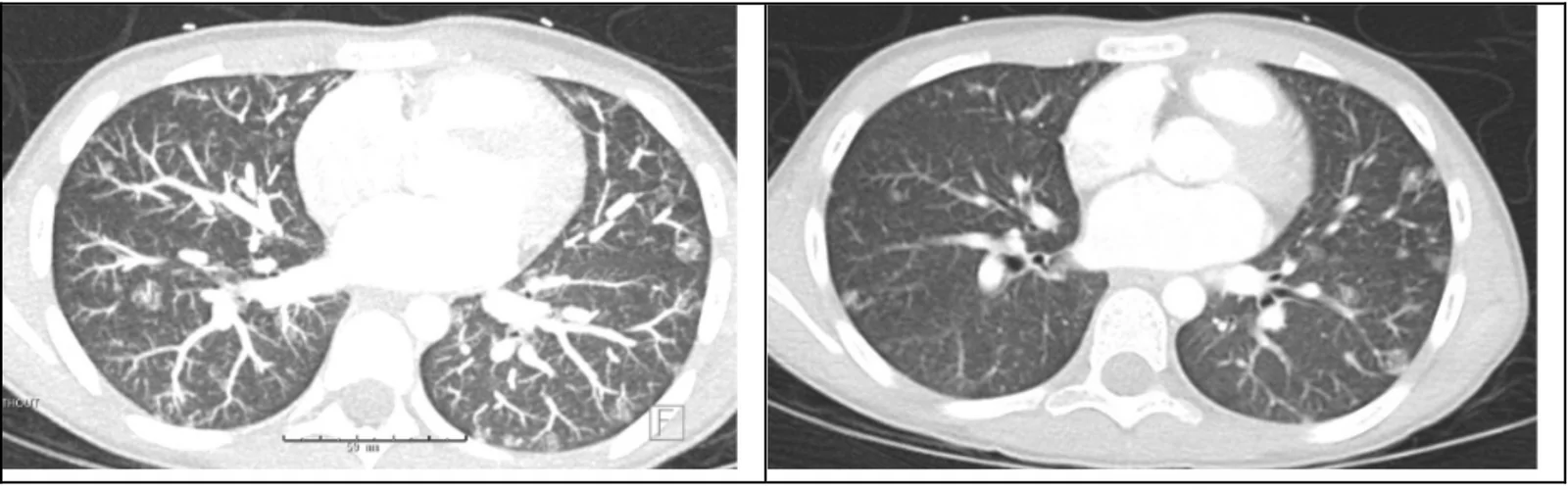
Chest CT (axial view) highlighting pulmonary nodules.

The patient was initiated on intravenous (IV) ceftriaxone 1 g (∼50 mg/kg) every 12 h, IV metronidazole 220 mg (10 mg/kg) every 8 h, IV dexamethasone 4 mg (0.15 mg/kg) every 8 h, and IV levetiracetam 220 mg (10 mg/kg) every 12 h. However, despite 2 weeks of antibiotic therapy, her brain lesions and lung nodules did not decrease in size. In fact, the brain lesions appeared to have increased in size, possibly due to the transient discontinuation of steroids (Figure 1). She eventually developed mild left upper-extremity weakness; otherwise, her neurologic exam remained unremarkable.

Given the presence of digital clubbing, refractory hypoxemia, and persistent diffuse pulmonary nodules despite antibiotic treatment, the patient was transferred to another children's hospital for further subspecialty evaluation, where concern persisted for an underlying shunt. Repeat transthoracic echocardiography with saline contrast demonstrated no intracardiac shunting. A lung perfusion scan revealed asymmetric perfusion with findings concerning for pulmonary shunting. Subsequent CT angiography of the chest confirmed diffuse PAVMs. The diffuse, small-caliber nature of the lesions initially precluded embolization; however, she later underwent embolization of four large PAVMs with interventional radiology.

A genetics consultation resulted in a recommendation for whole-genome sequencing. The patient was found to be heterozygous for a *de novo SMAD4* variant (c.1572G > T; p.Trp524Cys), a rare missense mutation previously reported in individuals with SMAD4-associated HHT/juvenile polyposis syndrome and currently classified in ClinVar as likely pathogenic, although additional data are needed to confirm this classification.

The patient remained on a prolonged course of antibiotics and oxygen support via nasal cannula and underwent three additional burr-hole drainage procedures with neurosurgery. However, serial imaging continued to demonstrate persistent intracranial abscesses. She was ultimately transferred to an HHT specialty center for ongoing multidisciplinary care and surveillance, where sirolimus was initiated. Her long-term course was further complicated by provoked seizures requiring antiepileptic therapy, as well as significant emotional and psychological distress. Figure 3 outlines the chronological sequence of events from the initial urgent care visit to evaluation and management at a tertiary children's hospital.

**Figure 3:**
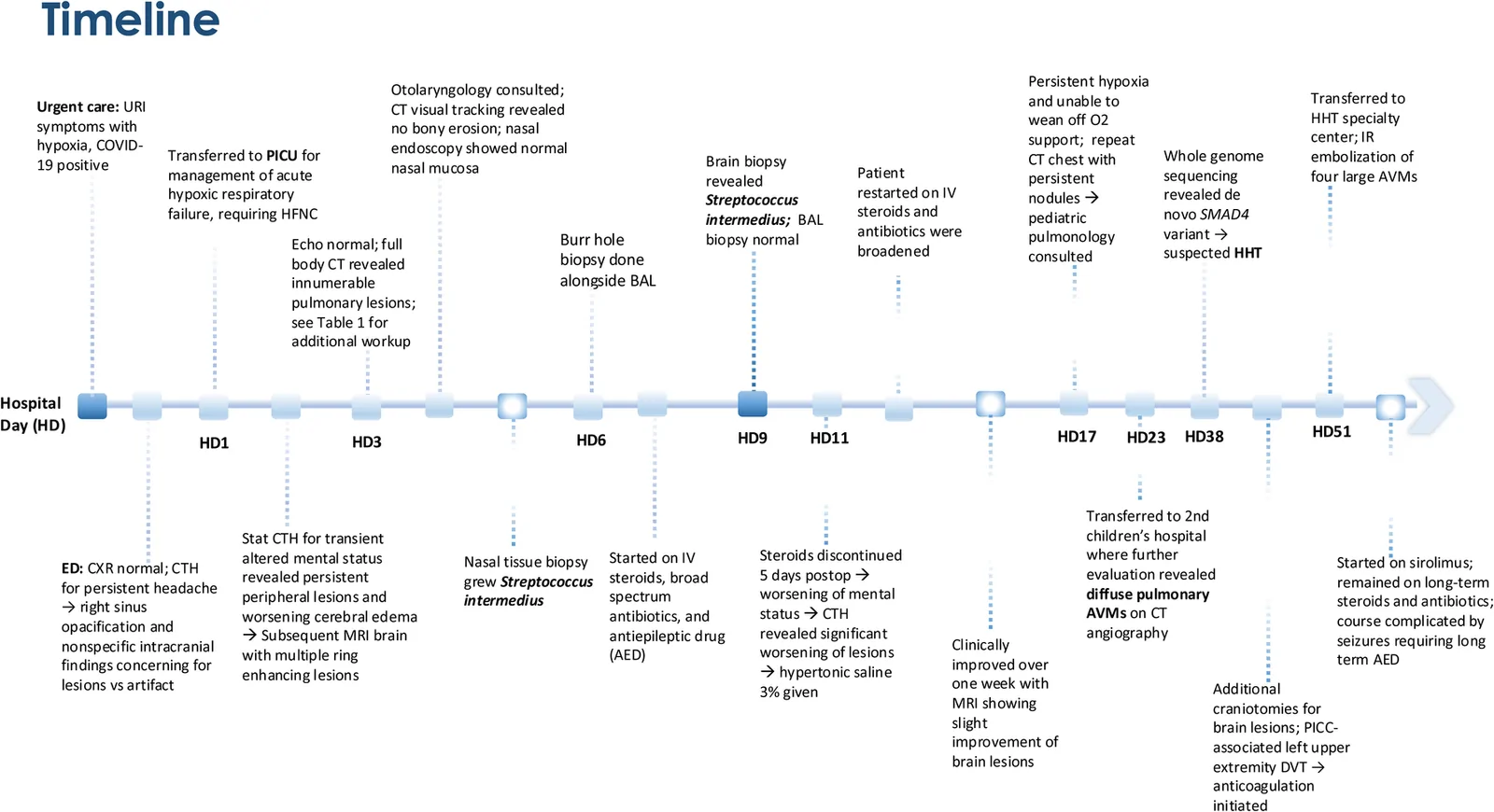
Timeline depicting key events in the patient's clinical course. AVMs, arteriovenous malformations; BAL, bronchoalveolar lavage; COVID-19, coronavirus disease 2019; CT, computed tomography; CTH, computed tomography of the head; CXR, chest X-ray; DVT, deep vein thrombosis; Echo, echocardiogram; HFNC, high-flow nasal cannula; HHT, hereditary hemorrhagic telangiectasia; IR, interventional radiology; IV, intravenous; MRI, magnetic resonance imaging; O_2_, oxygen; PICU, pediatric intensive care unit; URI, upper respiratory illness.

## Discussion

### HHT, JP/HHT overlap syndrome, and genetic findings

HHT is a genetic disease, typically inherited via an autosomal dominant trait, and is characterized by loss-of-function variants in genes encoding components of the TGF*β*/BMP signaling pathways (11). Although the majority of disease-causing variants in HHT are in the genes encoding *ENG* and *ACVRL1*, *SMAD4* mutations account for fewer than 5% of cases. In a retrospective study of 142 patients with genetically confirmed HHT, only 4% were found to have an *SMAD4* mutation, and among them, only one patient had both PAVMs and a cerebral abscess (12, 13).

Clinical diagnosis of HHT is established when an individual meets at least three of the Curaçao criteria: (1) family history of HHT, (2) recurrent epistaxis, (3) telangiectasias, and (4) visceral AVMs (14). However, the Curaçao criteria can be insufficient in diagnosing children due to the age-dependent development of some of these manifestations. In a retrospective multicenter study of 290 patients aged 0–21 years, the criteria had a sensitivity of 68% and specificity of 98%, with sensitivity being lowest in children aged 0–5 years. Our patient did not meet these criteria at presentation, as she had no family history and did not have a history of recurrent epistaxis or telangiectasias. For patients aged 0–21 who meet one or two criteria, genetic testing is the preferred method to confirm the diagnosis (14, 15).

Whole-genome sequencing was therefore critical in establishing the diagnosis in our patient. She was found to have a *SMAD4* missense mutation predicted to change tryptophan-524 to cysteine. This variant localizes to the MH2 domain of the Smad4 protein, which mediates interactions with transcription factors, including other Smad family proteins, and is a region in which many disease-associated mutations have been found. Identification of a pathogenic *SMAD4* variant in our patient not only confirmed an underlying HHT spectrum disorder but also established a diagnosis of JP/HHT overlap syndrome. In JP/HHT, 80% of disease-causing variants are in the MH2 domain of Smad4. A genotype–phenotype correlation has not been demonstrated in the literature, but this may be due to the rarity of the *SMAD4* variant in HHT. While the precise effects of the substitution in our patient are not known, a pathogenic variant changing tryptophan-542 to lysine has been described (16–18).

*SMAD4* functions within the TGF*β*/BMP signaling pathway and is uniquely associated with both HHT and juvenile polyposis syndrome, with varying degrees of phenotype observed in affected individuals (6). This phenotypic variability was well illustrated by our patient; although she carried a *SMAD4* variant associated with JP/HHT syndrome, she exhibited only HHT-related manifestations, specifically arteriovenous malformations, without clinical evidence of JP at the time of presentation. Notably, the same variant has been reported only once in ClinVar (Variation ID: 2147050), and that individual exhibited a phenotype consistent exclusively with juvenile polyposis syndrome (19). This contrast highlights both the rarity of our patient's presentation and the phenotypic variability associated with this syndrome, which may also explain why she did not meet the Curaçao criteria. Consistent with this phenotypic variability, a retrospective case series of 22 individuals with genetically confirmed JP/HHT overlap syndrome found that they were at high risk for findings from both syndromes to varying degrees, specifically epistaxis (77%), PAVMs (45%), and GI complications, including lower GI polyps (86%) and upper GI polyps (68%). Pertinently, 18% of individuals had no family history of HHT, and the median Curaçao score for all individuals was 3 (20).

### Predisposition to brain abscesses

Existing literature consistently demonstrates that patients with HHT, particularly those with PAVMs, are at increased risk for neurologic complications such as cerebral abscesses (1–4). One case–control study in the United Kingdom demonstrated that the risk of cerebral abscesses is 30 times greater in HHT individuals compared with controls (21). In a prospective study at an HHT center in Denmark, it was noted that among individuals with HHT and PAVMs, the prevalence of cerebral abscess was 7.8% (1).

Furthermore, many patients with underlying PAVMs are not diagnosed until after a cerebral abscess has been identified. In a retrospective questionnaire study of 233 patients with HHT, they found a 25.7-year delay between symptom onset and diagnosis (22). In one prospective study, 29 of 37 (78.4%) patients with a cerebral abscess and confirmed PAVMs had no PAVM diagnosis prior to their abscess diagnosis (23). A similar diagnostic delay to that of our patient was reported in a 3-year-old child whose unexplained brain abscess ultimately led to the discovery of an underlying PAVM and a new diagnosis of HHT (24). These findings, together with our case, reinforce the importance of evaluating for PAVMs and their underlying causes in children with unexplained brain abscesses where HHT features may not yet be evident.

Moreover, the classical brain abscess triad of findings includes headache, fever, and focal neurologic deficits. However, many studies show that this classical triad is often absent, making initial diagnosis challenging (25, 26). One retrospective cohort study including 27 children hospitalized with brain abscesses demonstrated that only 15% of patients had the classical triad of fever, headache, and neurologic deficits (27). This phenomenon may be explained by abscesses located in “silent” brain regions, which produce minimal or no focal symptoms until lesion enlargement or increasing surrounding edema occurs (28). Despite the presence of multiple brain abscesses, our patient demonstrated no focal neurologic deficits on initial presentation. Notably, the existing literature on brain abscess in HHT has focused primarily on incidence, risk factors, and outcomes, with limited characterization of lesion multiplicity (single vs. multiple) at the time of diagnosis.

In a recent 2026 cohort study looking at severe infections requiring hospitalization in 4,502 adult patients with HHT, 51 episodes of brain abscesses were reported among 43 patients. In these patients, the median length of antibiotic treatment was 52 days, and the most common pathogens were oral cavity isolates, primarily the *S. anginosus* group (*n* = 25, 58%). Furthermore, the study revealed that patients with HHT are at risk for other severe infections (including bacteremia, bone/joint infections, endocarditis, intraabdominal infections, and skin/soft-tissue infections), suggesting that they may have a weakened immune response and may potentially benefit from prophylactic strategies (29). Supporting this idea, experimental data in mice provide a potential mechanism for infectious predisposition in patients with HHT. Mice with deficient expression of the endoglin gene (*ENG*) in macrophages, a mutation known to cause HHT, showed a tendency to develop infections with opportunistic bacteria, predominantly *Staphylococcus aureus (*30).

In addition, alterations in T-lymphocyte phenotype have been reported in patients with HHT, suggesting that broader immune dysregulation may contribute to higher infection risk (31). These findings raise the possibility that both vascular abnormalities and impaired immune defense mechanisms may contribute to the development of brain abscesses. In our case, the isolation of *S. intermedius*, a member of the *S. anginosus* group, is consistent with the microbiologic profile previously reported in HHT-associated brain abscesses.

### Radiographic features and differential diagnosis

Ring-enhancing lesions identified on brain imaging in children usually include a broad differential, including pyogenic abscesses, inflammatory or autoimmune conditions [such as acute disseminated encephalomyelitis (ADEM)], parasitic infections (such as neurocysticercosis or toxoplasmosis), fungal infections known to have cerebral involvement (such as *Blastomyces*, *Cryptococcus*, or *Aspergillus*), or metastatic lesions (10, 32). Pertinently, the extent of disease in our patient, evident by multiple abscesses rather than one lesion, is less commonly reported in the literature. Typically, multiple abscesses are associated with hematogenous spread (secondary to endocarditis or right-to-left shunting), underlying immunocompromised status, or delayed management of an infection (10, 28).

Differentiating among the various etiologies required careful integration of clinical presentation, radiographic features, and diagnostic studies. Our patient's initial MRI identified multiple hemorrhagic ring-enhancing lesions with restricted diffusion and surrounding vasogenic edema, findings that are classically suggestive of intracranial abscesses. Pyogenic abscesses typically demonstrate thin, smooth ring enhancement with significant diffusion restriction [hyperintense on diffusion-weighted imaging (DWI) with low apparent diffusion coefficient (ADC) values] due to their purulent contents, differentiating them from inflammatory lesions or necrotic masses, which usually demonstrate facilitated diffusion (hypointense on DWI with high ADC levels) due to containing less viscous fluid (33–35).

The presence of multiple lesions with surrounding vasogenic edema and the location of lesions at the gray-white matter junction also raised concern for metastatic disease; however, in the absence of a primary malignancy, metastatic disease was unlikely. Moreover, fungal and parasitic infections were considered given the lesions’ characteristics and pulmonary involvement; however, these infections are often associated with immunosuppression or specific environmental exposures, which our patient did not have (32). ADEM was also considered, given that our patient presented with multifocal brain lesions in the setting of a viral infection; however, the radiographic features usually present with poorly defined, non-enhancing or incompletely enhancing lesions without restricted diffusion (36). Lastly, a direct contiguous spread was also considered given the presence of chronic sinusitis with isolation of the same organism; however, several features argued against this mechanism: the multifocal, bilateral distribution of abscesses (rather than solitary lesions in predictable locations), the absence of bony erosion/osteitis on sinus imaging, and notably her refractory hypoxemia and digital clubbing (37). These findings, combined with innumerable pulmonary nodules on chest CT, led to the diagnosis of diffuse PAVMs and ultimately juvenile polyposis/HHT overlap syndrome. In addition, the concordance between the bacterial species isolated from the brain abscess cultures and nasal swab culture raised suspicion that the patient's history of chronic sinusitis likely facilitated *S. intermedius* bacteremia, with subsequent and persistent shunting through the PAVMs and hematogenous seeding to the brain.

### Abscess expansion despite antibiotics

In our patient, the abscesses continued to enlarge despite appropriate coverage. This paradoxical worsening may be explained by several underlying pathophysiologic mechanisms. Recent research has demonstrated that cerebral abscesses may expand through osmosis secondary to neutrophil cell death. In one cross-sectional study on 47 patients who underwent repeated brain MRI without undergoing immediate pus drainage, the abscesses increased in all but two cases at a median rate of 23% increase per day despite antibiotic therapy and cultures showing no viable bacteria (38). Moreover, abscess infections can also persist despite appropriate antibiotics due to inadequate central nervous system penetration, where the abscess capsule and surrounding edema can impair delivery of the drug. Moreover, a recent 2026 cohort study reported that 14% of patients with PAVMs who underwent embolization still had recurrent brain abscesses (29).

## Conclusion

This case illustrates an atypical presentation and unusually early diagnosis of juvenile polyposis/HHT overlap syndrome due to a *SMAD4* variant, prompted by the incidental finding of multiple subclinical brain abscesses. PAVMs and genetic evaluation for HHT should be considered in pediatric patients with unexplained or multifocal brain abscesses, even when the Curaçao criteria are not met, as early recognition enables appropriate surveillance and prevention of neurologic complications.

## Data Availability

The raw data supporting the conclusions of this article will be made available by the authors without undue reservation.
